# Age-Dependent Effects of COVID-19 Vaccine and of Healthcare Burden on COVID-19 Deaths, Tokyo, Japan 

**DOI:** 10.3201/eid2809.220377

**Published:** 2022-09

**Authors:** Yura K. Ko, Hiroaki Murayama, Lisa Yamasaki, Ryo Kinoshita, Motoi Suzuki, Hiroshi Nishiura

**Affiliations:** National Institute of Infectious Diseases, Tokyo, Japan (Y.K. Ko, R. Kinoshita, M. Suzuki);; Tohoku University, Miyagi, Japan (Y.K.Ko);; International University of Health and Welfare, Chiba, Japan (H. Murayama);; University of Tokyo, Tokyo (L. Yamasaki);; Nagasaki University, Nagasaki, Japan (L. Yamasaki); Kyoto University, Kyoto, Japan (H. Nishiura)

**Keywords:** COVID-19, 2019 novel coronavirus disease, coronavirus disease, severe acute respiratory syndrome coronavirus 2, SARS-CoV-2, viruses, respiratory infections, zoonoses, vaccine effectiveness against death, case-fatality risk, healthcare burden, vaccines, Japan

## Abstract

COVID-19 vaccine effectiveness against death in Japan remains unknown. Furthermore, although evidence indicates that healthcare capacity influences case-fatality risk (CFR), it remains unknown whether this relationship is mediated by age. With a modeling study, we analyzed daily COVID-19 cases and deaths during January–August 2021 by using Tokyo surveillance data to jointly estimate COVID-19 vaccine effectiveness against death and age-specific CFR. We also examined daily healthcare operations to determine the association between healthcare burden and age-specific CFR. Among fully vaccinated patients, vaccine effectiveness against death was 88.6% among patients 60–69 years of age, 83.9% among patients 70–79 years of age, 83.5% among patients 80–89 years of age, and 77.7% among patients >90 years of age. A positive association of several indicators of healthcare burden with CFR among patients >70 years of age suggested an age-dependent effect of healthcare burden on CFR in Japan.

Since the emergence of SARS-CoV-2 in Japan, COVID-19 has remained a substantial public health concern. As of September 1, 2021, the cumulative number of reported cases in Japan had reached 1,482,000, resulting in >16,000 deaths. Among those, 346,742 cases and 2,500 deaths occurred in Tokyo alone (*1*). Officials at various levels have requested the cooperation of residents in implementing several nonpharmaceutical measures to mitigate disease burden and prevent the collapse of the healthcare system (*2*). In addition, mass vaccination has been publicly available in Japan since April 12, 2021, initially targeting persons >65 years of age. Three types of vaccine have been used in Japan: mRNA-1273 (Moderna, https://www.modernatx.com), BNT162b2 vaccine (Pfizer-BioNTech, https://www.pfizer.com), and ChAdOx1 nCoV-19 vaccine (AstraZeneca, https://www.astrazeneca.com). As of September 1, 2021, vaccination coverage in Tokyo reached 58.1%, and 87.9% of persons >65 years of age had completed the 2-dose regimen (*3*).

Numerous studies have investigated vaccine effectiveness (VE) against SARS-CoV-2, and most derived their estimates by using test-negative designs or cohort analyses (*4*–*7*). However, attaining a sample size sufficient for such rigorous epidemiologic investigation requires a substantial amount of time, especially in countries like Japan, where COVID-19 mortality rate is low. Nonetheless, the need for valid real-world estimates of VE against death is urgent.

Case-fatality risk (CFR) acts as a proxy for disease virulence and is often used to identify risk factors for death within a population (*8*). A previous study indicated that the CFR for COVID-19 varies substantially with age (*9*). Detailed time-series surveillance data, including incidence and death information as well as background characteristics (e.g., patient age, sex, vaccination status), are critical for elucidating SARS-CoV-2 virulence in real time during the pandemic (*9*). Therefore, we aimed to jointly estimate VE against death and CFR on the date of death according to age by using both local-level and national-level surveillance data from Tokyo during the period when the Alpha or Delta variant was predominant (*10**,**11*). Crude CFR, defined as the ratio of the cumulative number of deaths to the cumulative number of confirmed cases, can underestimate actual CFR when cases are increasing and overestimate it when they are decreasing (*12*). To address this issue, which results from the delay from reporting to death and is referred to as right truncation bias, we constructed a mathematical model incorporating the time delay.

Several studies have also demonstrated that healthcare capacity substantially influences CFR (*13*–*15*). As COVID-19 incidence increases, the healthcare burden in the region also increases, and infected persons with severe signs/symptoms may not be able to receive rapid and appropriate treatment (i.e., ventilator support, extracorporeal membrane oxygenation [ECMO]). Thus, CFR may increase, given that a limited number of patients with severe signs/symptoms would be able to receive proper treatment. Therefore, monitoring healthcare capacity along with CFR could be a way to implement appropriate public health interventions tailored to the temporal situation. With this study, we aimed to examine temporal changes in age-specific CFR with respect to the healthcare burden. No ethics approval was required because the data were anonymized and collected in response to the outbreak.

## Methods

### Data Collection

In this modeling study, we used publicly available local-level data published by the Tokyo Metropolitan Government (*16*) to estimate VE against death and time-varying CFR. The number of positive cases according to the date of diagnosis and the number of deaths at specific time points were evaluated for each age group. We obtained data regarding vaccination status (fully vaccinated, partially vaccinated, and unvaccinated) among SARS-CoV-2–positive persons and deaths in Tokyo during each epidemiologic week by using the national-level Health Center Real-Time Information-Sharing System on COVID-19 (HER-SYS) (*17*).

The government of Japan recognized COVID-19 as a designated infectious disease in January 2020, after which a legal mandate to report all confirmed COVID-19 cases to the Ministry of Health, Labour, and Welfare was issued. In Tokyo, in addition to PCR and antigen testing for symptomatic persons and close contacts, screening testing has been conducted to detect outbreaks at early stages in facilities where clusters are likely to occur (*18*). Since May 2020, incidence reporting to the national government has been conducted through HER-SYS, and all diagnosed COVID-19 cases in the country are registered in this system. Although essential epidemiologic information for case identification (e.g., age, sex, and date of diagnosis) is entered into HER-SYS, details regarding prognosis may be incomplete. Thus, information related to death may not be entered, especially when public health centers are faced with an overwhelming workload. However, as a public health measure, the Tokyo Metropolitan Government has published information related to all COVID-19 deaths in Tokyo, including age, date of diagnosis, and date of death.

To analyze the effect of the healthcare burden on the CFR, we obtained the daily proportion of asymptomatic cases at the time of report in each age group by using HER-SYS. We collected data associated with the following 4 variables from the Tokyo Metropolitan Government website and used them as indicators of the healthcare burden in Tokyo (*19*): the number of persons with severe COVID-19 cases, defined as those requiring a ventilator or ECMO; the number of persons to whom the Tokyo rules apply, defined as persons for whom the care site destination has not been determined within 20 minutes of the emergency medical services team’s request for or selection of 5 medical institutions to receive the patient; the proportion of nonhospitalized COVID-19 case-patients among all case-patients reported that day; and the proportion of case-patients for whom coordination of the care site was in progress.

To avoid underestimating the disease burden of COVID-19, we defined COVID-19-related deaths as death within 60 days of diagnosis, not within 28 days. To account for reporting delays, we accessed data as of November 15, 2021, for both HER-SYS and Tokyo Metropolitan Government published data, and we included data up to August 31, 2021.

### Handling of Vaccination Data

For case-patients for whom date of vaccination was known, we considered those for whom date of vaccination and date of diagnosis were separated by 14 days to be fully vaccinated. All case-patients for whom date of vaccination was unknown were also considered fully vaccinated.

To avoid underestimating the number of positive cases and deaths among fully vaccinated case-patients, we used logistic regression to impute vaccination status for case-patients with unknown vaccination history in HER-SYS. In this analysis, we considered the month of diagnosis, age, presence of symptoms, and occurrence of death to be associated with vaccination history (Appendix).

### Estimating VE Against Death and Time-Varying CFR According to Age Group

To jointly estimate VE against death and CFR by age group, we modeled the process to generate data for deaths by using data for confirmed case-patients for whom date of diagnosis was available and for deceased case-patients for whom date of death was available. Because a delay between diagnosis and death leads to complexity when using confirmed cases and deaths in a model (Appendix Figure 1), we convoluted the incidence on a specific date of diagnosis with the function representing the age-specific relative frequency of the delay and estimated the number of cases on a provisional date of death as a denominator of CFR. In this setting, we explicitly modeled the binomial process according to vaccination history while incorporating VE and CFR against death for both partially and fully vaccinated case-patients, which were considered unknown parameters. We estimated these unknown parameters by using the Markov chain Monte Carlo method with improper flat priors. In addition, we calculated the probabilities representing the unconditional protection against death for partially and fully vaccinated persons by exploiting the posterior distributions of VE against death and VE against documented infection in Japan, as reported elsewhere (*20*). We conducted all procedures according to age stratification (Appendix).

To determine the effect of COVID-19 vaccination on the burden of the disease, we simulated the daily cumulative incidence of deaths by using the estimated conditional VE against death and CFR in 2 scenarios. The scenarios were if all reported case-patients were fully vaccinated and if all positive case-patients had not been fully vaccinated.

As a sensitivity analysis, we also estimated VE and CFR against death in 2 contexts. First, we excluded all case-patients with unknown vaccination status and definition of death occurring within 28 days of diagnosis as COVID-19–related. In addition, to avoid overestimation, we used the different assumptions of VE against documented infection, setting 40% for partially vaccinated case-patients and 80% for fully vaccinated case-patients on the basis of previous reports, which estimated VE against infection with the Delta variant (*4*).

### Effects of Healthcare Burden on CFR, According to Age Group

Next, we explored the effect of the healthcare burden on time-varying age-specific CFR by using data for confirmed case-patients and deaths of unvaccinated case-patients. We constructed a Bayesian multilevel model for the unvaccinated population based on the binomial process, which had already been constructed for the joint estimation of CFR and VE. Assuming that the CFR follows a β distribution in the binomial process, we performed an inverse logit transformation on its mean, in which the explanatory variables were embedded as a regression model. As explanatory variables, we considered the daily empirical asymptomatic rate by age and each of the 4 healthcare burden indicators. The asymptomatic rate was added as an explanatory variable because that rate may be considered to be biologically identical among the infected population; however, the rate among PCR-confirmed case-patients varies because of changes in testing policies and intensity of contact tracing (ascertainment bias) and, therefore, is suitable for adjusting for such bias.

We then selected the best-fit model for the length of lag of each healthcare burden indicator. We estimated the parameters (i.e., coefficients α_1_ and α_2_, intercept β, and the variance of the β distribution κ) in a Bayesian framework with weakly informative priors (α_1_, β_2_, and β are normally distributed with mean 0 and variance 100; κ is normally distributed with mean 0 and variance 10). For model selection, we computed the widely applicable information criterion by using the marginalized posterior log-likelihoods (*21*). We used Markov chain Monte Carlo for this analysis (Appendix).

## Results

**Figure 1:**
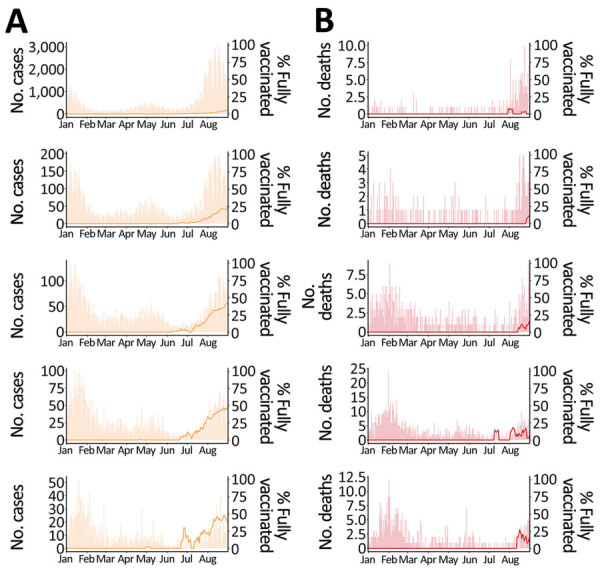
Effects of COVID-19 vaccine on deaths, stratified by age group, Tokyo, Japan, January–August 2021. A) Daily number of SARS-CoV-2–positive case-patients (bar) and proportion of fully vaccinated SARS-CoV-2–positive case-patients (line, 7-day moving average). B) Daily number of deaths (bar) and proportion of deaths among fully vaccinated case-patients (line, 7-day moving average). Ranges indicate years of age. From top to bottom, age groups are 30–59, 60–69, 70–79, 80–89, >90 years.

We included in our study all 2,065 COVID-19–associated deaths reported by the Tokyo Metropolitan Government for case-patients >30 years of age during January 1–August 31, 2021. We calculated the percentages by vaccination status for each of SARS-CoV-2–positive case-patients and deaths by using HER-SYS data, which required imputation of unknown vaccination status for 10,327 case-patients. On the basis of imputation, we considered 752 case-patients to be partially vaccinated, 390 fully vaccinated, and the rest unvaccinated. The proportions of positive cases and deaths after full vaccination increased over time, starting with older adults (Figure 1). 

Among partially vaccinated case-patients, estimates of VE against death in each age group ranged from 34% to 66%. Among fully vaccinated case-patients, VE against death were 38.0% (95% credible interval [CrI] 2.6–82.4), 88.6% (64.3–98.1), 83.9% (68.8–92.9), 83.5% (72.5–91.0), and 77.7% (60.7–89.4) among case-patients who were 30–59, 60–69, 70–79, 80–89, and >90 years of age, respectively. Unconditional protection against death was estimated as 93.8% (90.3–98.2) in the 30–59-year age group and >97% in the >60-year age groups (Table). The sensitivity analysis revealed similar results for age-specific VE (Appendix Table 1). We also observed temporal changes in CFR according to age group. Minimum estimated median values were 0.14%, 1.30%, 3.12%, 6.81%, and 8.87% and maximum estimated median values 1.02%, 5.37%, 13.76%, 27.08%, and 41.16% among persons 30–59, 60–69, 70–79, 80–89, and >90 years of age, respectively (Appendix Figure 2).

**Table:** Estimated COVID-19 vaccine effectiveness in terms of protection against death over test-positive and unconditional protection against death, by age group, Tokyo, Japan, January 1–August 31, 2021*

Effect of interest, by age, y	Vaccine effectiveness (95% credible interval)	
	Partially vaccinated*	Fully vaccinated†
Protection against death over documented infection		
30–59 y	34.2 (2.2–71.4)	38.0 (2.6–82.4)
60–69 y	66.1 (33.0–85.4)	88.6 (64.3–98.1)
70–79 y	38.2 (7.3–63.8)	83.9 (68.8–92.9)
80–89 y	46.4 (17.9–68.7)	83.5 (72.5–91.0)
>90 y	52.7 (19.6–76.6)	77.7 (60.7–89.4)
Unconditional protection against death		
30–59 y	68.7 (53.5–86.4)	93.8 (90.3–98.2)
60–69 y	83.9 (68.1–93.0)	99.2 (97.4–99.9)
70–79y	70.6 (55.9–82.8)	99.3 (98.6–99.7)
80–89 y	74.5 (60.9–85.1)	99.4 (98.9–99.6)
>90 y	77.5 (61.8–88.8)	98.4 (97.1–99.2)

*14 d after the first vaccination and <14 d after the second vaccination. †14 d after the second vaccination.

**Figure 2:**
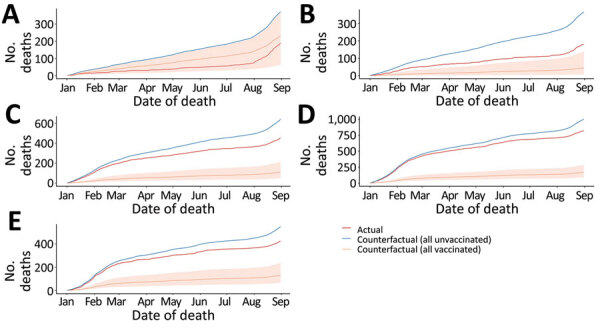
Cumulative number of COVID-19 deaths in counterfactual scenarios (i.e., all positive case-patients vaccinated, all positive case-patients unvaccinated, and actual reported cases), Tokyo, Japan, January 1–August 31, 2021. A) 30–59; B) 60–69; C) 70–79; D) 80–89; E) >90 years of age. Shaded areas represent 95% credible intervals

Subsequently, our simple simulation showed that among case-patients >60 years of age, if all had been vaccinated, the counterfactual number of deaths would have been less than one third to one fifth of actual deaths. If all case-patients had been unvaccinated, the counterfactual number of deaths would have increased by 100–200 in each age group (Figure 2).

**Figure 3:**
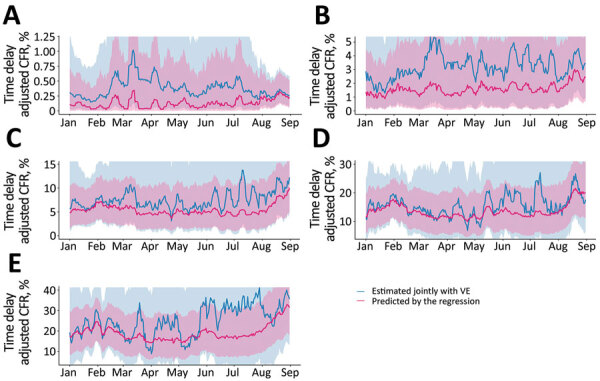
Comparison of COVID-19 CFR predictions based on indicators of healthcare burden and asymptomatic rate with CFR when jointly estimated with VE, Tokyo, Japan, January 1–August 31, 2021. Shaded areas show 95% credible intervals. A) 30–59; B) 60–69; C) 70–79; D) 80–89; E) >90 years of age. CFR, case-fatality risk; VE, vaccine effectiveness.

An exploratory analysis of the effect on CFR of each of the 4 Tokyo healthcare burden indicators according to age group revealed that the number of severe cases was positively associated with CFR for persons 70–79, 80–89, and >90 years of age. Likewise, the number of cases to which Tokyo rules applied also exhibited a positive association with CFR among those who were 70–79 years of age, and both the proportion of nonhospitalized case-patients and the proportion of case-patients for whom coordination of hospital care was in progress were positively associated with CFR among persons who were 80–89 years of age (Appendix Table 2). When we compared CFR estimated using the best-fit model with CFR estimated jointly with the VE against death, the models in the 70–79-year and 80–89-year age groups captured a part of the variation in the temporal change in CFR (Figure 3). We were not able to capture the time variation in CFR and the healthcare burden indicators for those in the 30–69-year age groups.

## Discussion

Our joint estimation of VE against death and age-specific CFR in Japan, using surveillance data, indicated that during the period when the SARS-CoV-2 Alpha or Delta variant was predominant (*10*,*11*), VE against death in fully vaccinated persons >60 years of age was as high as 80%; estimates were unstable for those 30–59 years of age because of the small number of deaths in the corresponding age groups. Unconditional protection from death was also estimated to be extremely high (>97%) for persons in all age groups. These results suggest that even if breakthrough infections occur, VE against deaths was sufficiently high enough to avert most of the risk for COVID-19 death during the predominant periods of Alpha and Delta. Given the length of time required to collect sufficient cases to achieve a plausible estimate of VE against death in test-negative designs or cohort studies, our analysis of surveillance data demonstrates the benefits of vaccine dissemination in the general population. We also believe that the approach used in our study, which jointly estimated CFR and VE against death by using only the number of positive cases and deaths according to vaccination status, is a practical method that can be naturally extended to the effectiveness of booster vaccine and applied in various countries and regions.

Our results suggest that VE against death from COVID-19 declines with age. These results are consistent with the findings of previous reports, which indicated that the decline in VE estimates observed in older adults may be related to age-related immune senescence, which limits the protective effects of vaccines (*22*).

In the exploratory analysis investigating the effect of the healthcare burden on CFR, the regression coefficients with their uncertainty bounds were positive for many indicators among older adults, especially those 80–89 years of age, including the number of patients with severe cases, the proportion of nonhospitalized case-patients, and the proportion of case-patients for whom coordination of the care site was in progress. Among those 70–79 and 80–89 years of age, the healthcare burden indicators and rate of asymptomatic illness captured the upward trend in CFR in February and August 2021; among those >90 years of age, some of the time-varying trends were captured, although they frequently diverged from the estimated CFR. This finding may be because do-not-resuscitate orders are relatively more common among persons >90 years of age, which may have been an unconsidered confounder in our analysis. Furthermore, the temporal change in CFR could not be captured by the healthcare burden indicators for the younger age groups. Although many countries have reported that mortality rate increases with healthcare burden and reduced capacity (*13*–*15*), our results suggest that the effect of healthcare burden on CFR differs according to age group. One explanation is the possibility that medical resources such as ventilators and ECMO were preferentially allocated to young persons with severe cases. As in France in the early stages of the pandemic (*23*), even if not explicitly documented, these triage procedures probably took place, especially during July–August 2021, when the number of infected persons and deaths soared. From a clinical perspective, delays in treatment initiation have been reported to lead to poor outcomes (*24*). It is also possible that frail older case-patients were more affected by delays in treatment initiation because of healthcare burden. Such age-dependent differences may be useful for determining appropriate public health countermeasures. However, as we aimed to propose that vulnerability to healthcare burden varies by age, the effect of healthcare burden may not necessarily be causal. Further studies are required to determine whether this hypothesis can be constructed with causality.

Among the limitations of our study, we obtained information about vaccination status from HER-SYS. During the study period, vaccination status in the HER-SYS was automatically entered as unvaccinated if no input was provided, which may have resulted in underestimation of case-patients with unknown vaccination status. However, the proportion of fully vaccinated case-patients increased over time as the vaccination coverage in the population progressed (Figure 1). Furthermore, the same trend was observed even in August 2021, when the epidemic was most severe and the workload at public health centers and hospitals was considered the greatest, suggesting that the quality of data entered for vaccination status was maintained to some extent. A second limitation is that although other risk factors, such as underlying disease, can contribute to severe outcomes, we were not able to take this into account because of the limited information available in the surveillance dataset. Differences in the proportions of these risk factors between the vaccinated and unvaccinated groups may have biased estimates of VE. Third, we could not obtain information about reinfection status from the dataset. However, the incidence of reinfection was reported to be very low before the emergence of the Omicron variant (*25*). We believe that the effect of reinfection on our analysis was small. Fourth, when exploring the effect of healthcare burden on CFR, we did not consider the effects of newly emerging variants. In Japan, during the study period, the Alpha variant began to increase in February 2021 and became predominant in June, and the Delta variant surged in the following 2 months (*10*). Fifth, we did not account for the introduction of therapeutic agents (antibody cocktail of casirivimab and imdevimab) in our analysis. Sixth, we used the number of persons with severe cases and the proportion of nonhospitalized case-patients as proxies for healthcare burden but did not take into account the increased provision of bed capacity for hospitalized patients and patients with severe cases during the study period. However, as with the other proxy indicators of healthcare burden used in our study, we believe that they indirectly reflect part of the medical situation because they also peaked during January–February and July–August 2021, when the number of cases increased (Appendix Figure 3). Last, ascertainment bias is always a problem when estimating CFR, and it may not have been fully eliminated in this analysis. Although testing policies (PCR or antigen tests) did not change substantially over the study period, the frequency and scale of screening tests may have been affected by the size of the epidemic. In particular, screening capacity for nursing homes was probably limited in August 2021, when the magnitude of the epidemic was greatest. This possibility is suggested by the fact that the proportion of asymptomatic case-patients among those >90 years of age decreased during the same period (Appendix Figure 4). However, we attempted to address this bias as much as possible by including the asymptomatic rate among SARS-CoV-2–positive case-patients in Tokyo as an explanatory variable.

In summary, our study estimated VE against death in Japan based on surveillance data. Our findings highlight the potential effect of the healthcare burden on CFR, especially among older adults. The positive association between several indicators of healthcare burden and CFR among patients >70 years of age suggests an age-dependent effect of healthcare burden on CFR in Japan.

Appendix Supplemental results for study of age-dependent effects of COVID-19 vaccine and of healthcare burden on COVID-19 deaths, Tokyo, Japan.
