# Supplementary material for: Age-Dependent Effects of COVID-19 Vaccine and of Healthcare Burden on COVID-19 Deaths, Tokyo, Japan
Source: Emerg Infect Dis. 2022 Sep;28(9):1777–84. doi: 10.3201/eid2809.220377 (PMC9423928; doi:10.3201/eid2809.220377)
Supplement: Appendix — Supplemental results for study of age-dependent effects of COVID-19 vaccine and of healthcare burden on COVID-19 deaths, Tokyo, Japan. [file 22-0377-Techapp-s1.pdf]

Article DOI: https://doi.org/10.3201/eid2809.220377

Age-Dependent Effects of COVID-19 Vaccine and of Healthcare Burden on COVID-19 Deaths, Tokyo, Japan

Appendix

Model specification

Handling missing data in vaccination status

We exploited logistic regression to impute the vaccination history of cases with unknown vaccination status according to the following equation, in which the month of diagnosis, age, presence of symptoms, and occurrence of death were considered associated with vaccination status.

$$Vacc\ status_i = logistic(z_i)$$

$$z_i = age_i + month_i + symptom_i + death_i \quad (2)$$

In the above equation, a vaccination status of 0.5 or more was regarded as vaccinated, while a status of less than 0.5 was regarded as unvaccinated. The calculation was performed using the "*MICE*" package in R version 4.0.3.

Joint estimation of age- and dose-specific vaccine effectiveness from death and case fatality risk

The empirical dataset consisted of the daily number of COVID-19 documented infection on a confirmed date and the daily number of deaths on a date of death by age group and vaccination status. Let $c_{a,x}(t)$ and $D_{a,x}(t)$ denote the number of cases with moving averages of

7 days and the number of deaths on a date of death in age group $a \in [0,1,2,3,4]$ (corresponding to age group 30–59, 60–69, 70–79, 80–89, 90 years and over) with vaccination status $x \in [0,1,2]$ (corresponding to "without vaccination", "partially vaccinated", and "fully vaccinated"). To address the right truncation bias caused by the time-delay from case confirmation to death, we projected the number of cases on a provisional date of death $c_{a,x}(t)$ out of the number of cases on a confirmed date $C_{a,x}(t)$:

$$C_{a,x} = MA(\sum_{\tau=0}^{t} c_{a,x}(t-\tau)f(\tau)) \tag{1}$$

where $f(\cdot)$ denotes the relative frequency of the time-delay from case confirmation to death. $f(\cdot)$ was obtained by fitting the empirical distribution derived from HER-SYS database and estimating the empirical cumulative distribution function $F(t)$. Then $f(t)$ is deemed to be $F(t) - F(t-1)$ for $t > 0$. $MA(\cdot)$ represents the function of moving averages of 7 days. We smoothed $C_{a,x}(t)$ and $D_{a,x}(t)$ with moving average of 7 days to alleviate week effects. In this setting, we constructed the process to generate deaths $D_{a,x}(t)$ out of $C_{a,x}(t)$ by assuming a binomial distribution and arrived at the likelihood function as:

$$L(\boldsymbol{\Theta}; \boldsymbol{D}) = \prod_{a}\prod_{t} Binom\left(D_{a,0}(t); C_{a,0}(t), p_{a,0}(t)\right) Binom\big(D_{a,1}(t); C_{a,1}(t), (1 - \epsilon_{a,1})p_{a,0}(t)\big) Binom\big(D_{a,2}(t); C_{a,2}(t), (1-\epsilon_{a,2})p_{a,0}(t)\big) \tag{2}$$

where $\boldsymbol{\Theta}$ and $\boldsymbol{D}$ represent the set of parameters and the set of data, respectively. $p_{a.0}(t)$, $\epsilon_{a,1}$, and $\epsilon_{a,2}$ denote case fatality risk (CFR) in age group a without vaccination on a provisional date of death t, the vaccine effectiveness against death for those who are partially vaccinated, and vaccine effectiveness against death for those who are fully vaccinated, respectively. Then CFR of those who are partially vaccinated and fully vaccinated is calculated as $(1-\epsilon_{a,1})p_{a,0}(t)$ and $(1-\epsilon_{a,2})p_{a,0}(t)$.

The set of parameters was estimated in a Bayesian framework. All of the parameters, $p_{a.0}(t)$, $\epsilon_{a,1}$, and $\epsilon_{a,2}$, are with improper flat priors. We employed dynamic Hamiltonian Monte Carlo (HMC) with No-U-Turn-Sampler (NUTS) as Markov chain Monte Carlo method (MCMC) and four chains of 1,000 thinned samples from 10,000 MCMC steps were obtained while the first 1,000 steps of the chain were discarded as a warm-up. MCMC method was implemented "*rstan*" package in R version 4.0.3.

In addition, the probabilities representing the unconditional protection against death of partially vaccinated or fully vaccinated were calculated by exploiting the posterior distributions of the vaccine effectiveness against death and VE against documented infection in Japan (*1*).

The impact of indicators of the level of healthcare burden on case fatality risk by age

We utilized the above-mentioned model for unvaccinated population to explore the impact of healthcare burden on CFR. We incorporated beta distribution into CFR $p_{a,0}(t)$ in the binomial process. we performed an inverse logit transformation on its mean in which explanatory variables are embedded as a regression model. As explanatory variables, we consider the daily empirical asymptomatic rate by age and each of the four healthcare burden indicators. The asymptomatic rate was added as an explanatory variable because the asymptomatic rate can be assumed to be biologically identical among the infected population, but the rate among PCR confirmed cases varies due to ascertainment bias, making it suitable for considering the effect of ascertainment bias. Then the model specification is given as:

$$D_{a,0} \sim Binom(C_{a,0}, p_{a,0}(t))$$
$$p_{a,0}(t) \sim Beta(mean = \mu_a(t), variance = \kappa_a) \quad (3)$$

where α_1, α_2, and β are two coefficients for the explanatory variables x_1 and x_2, and the intercept. Then the total likelihood is constructed as:

$$L(\mathbf{\Theta}; \boldsymbol{D}) = \prod_a Binom\left(D_{a,0}(t); C_{a,0}(t), p_{a,0}(t)\right) Beta(p_{a,0}(t); logit^{-1}(\alpha_1 x_1(t) + \alpha_2 x_2(t) + \beta), \kappa_a) \quad (4)$$

To estimate the set of parameters $\mathbf{\Theta}$ in the multilevel model, we employed HMC with NUTS and four chains of 1,000 thinned samples from 10,000 MCMC steps were obtained while the first 1,000 steps of the chain were discarded as a warm-up. We selected the best-fit lag for each indicator of healthcare burden by wildly applicable information criterion (WAIC) marginalizing the posterior log-likelihoods (*2*).

Appendix Table 1. The estimated vaccine effectiveness (protection against death over test-positive and unconditional protection against death) by age group as sensitivity analysis, excluding all cases with unknown vaccination status

		VE (95% CrI)	
Effect of interest	Age group	Partially vaccinated*	Fully vaccinated†
Protection against death	30–50s	52.8 (6.9–84.5)	30.5 (1.4–75.8)
over documented infection	60s	66.3 (33.6–85.0)	85.8 (60.8–97.1)
	70s	34.9 (5.0–60.4)	77.9 (61.0–89.0)
	80s	49.3 (22.2–69.7)	82.6 (70.8–90.2)
	90s+	55.3 (24.6–78.9)	76.4 (58.6–87.9)
Unconditional protection	30–50s	77.6 (55.7–92.6)	93.0 (90.1–97.6)
against death	60s	83.9 (68.4–92.9)	98.9 (97.1–99.8)
	70s	69.0 (54.8–81.1)	99.0 (98.2–99.5)
	80s	75.9 (62.9–85.6)	99.3 (98.9–99.6)
	90s+	78.7 (64.1–90.0)	98.3 (96.9–99.1)

*Partially vaccinated is defined as those who have been 14 days after the first vaccination and less than 14 days after the second vaccination.

†Fully vaccinated is defined as those who have been 14 days after the second vaccination.

Appendix Table 2. Estimated vaccine effectiveness (protection against death over test-positive and unconditional protection against death) by age group as sensitivity analysis when considering a COVID-19-related death as being within 28 days of the date of diagnosis to the date of death.

		VE (95% CrI)	
Effect of interest	Age group	Partially vaccinated*	Fully vaccinated†
Protection against death over documented infection	30–50s	33.8 (2.8–69.8)	38.9 (2.2–81.5)
	60s	63.8 (30.8–83.7)	88.1 (64.0–97.8)
	70s	36.8 (7.2–62.9)	84.3 (70.1–93.1)
	80s	45.8 (18.3–67.5)	82.6 (70.9–90.2)
	90s+	50.6 (20.6–74.1)	77.0 (60.0–88.6)
Unconditional protection against death	30–50s	68.5 (53.7–85.6)	93.9 (90.2–98.2)
	60s	82.8 (67.1–92.2)	99.1 (97.3–99.8)
	70s	69.9 (55.8–82.3)	99.3 (98.6–99.7)
	80s	74.2 (61.1–84.5)	99.3 (98.9–99.6)
	90s+	76.5 (62.2–87.7)	98.3 (97.0–99.2)

*Partially vaccinated is defined as those who have been 14 days after the first vaccination and less than 14 days after the second vaccination.

†Fully vaccinated is defined as those who have been 14 days after the second vaccination.

Appendix Table 3. Estimated vaccine effectiveness (protection against death over test-positive and unconditional protection against death) by age group as sensitivity analysis, assuming that VE against documented infection is 40% for partially vaccinated and 80% for fully vaccinated.

		VE (95% CrI)	
Effect of interest	Age group	Partially vaccinated*	Fully vaccinated†
Unconditional protection against death	30–50s	53.3 (31.7–80.1)	87.6 (80.4–96.4)
	60s	75.9 (52.4–89.5)	97.6 (92.2–99.6)
	70s	58.1 (36.4–75.6)	96.5 (93.3–98.5)
	80s	63.9 (44.4–79)	96.7 (94.3–98.1)
	90s+	67.9 (46.5–84.6)	95.1 (91.3–97.6)

* Partially vaccinated is defined as those who have been 14 days after the first vaccination and less than 14 days after the second vaccination.

†Fully vaccinated is defined as those who have been 14 days after the second vaccination.

Appendix Table 4. Summary of the estimated coefficient of each indicator of the level of healthcare burden*

Age group	Model	Coefficient	WAIC
30–50s	Model 1	0.001 (-0.001–0.003)	6.568
	Model 2	0.001 (-0.003–0.006)	6.567
	Model 3	0.110 (-1.362–1.554)	6.567
	Model 4	0.268 (-0.601–1.084)	6.569
60s	Model 1	0.002 (-0.000–0.004)	3.757
	Model 2	0.002 (-0.002–0.006)	3.755
	Model 3	-0.136 (-1.477–1.211)	3.753
	Model 4	0.207 (-0.631–1.042)	3.754
70s	Model 1	0.003 (0.001–0.005)	3.357
	Model 2	0.004 (0.000–0.007)	3.357
	Model 3	0.189 (-0.742–1.140)	3.357
	Model 4	0.561 (-0.047–1.182)	3.357
80s	Model 1	0.002 (0.001–0.004)	3.004
	Model 2	0.003 (-0.000–0.006)	3.004
	Model 3	0.740 (0.053–1.421)	3.004
	Model 4	0.736 (0.215–1.243)	3.004
90s+	Model 1	0.003 (0.001–0.005)	2.043
	Model 2	0.003 (-0.000–0.007)	2.043
	Model 3	0.785 (-0.069–1.689)	2.043
	Model 4	0.673 (-0.024–1.325)	2.043

*Model 1: The number of severe COVID-19 cases; Model 2: The number of people in which Tokyo rules applied; Model 3: The proportion of non-hospitalized COVID-19 cases; Model 4: The Proportion of COVID-19 cases in which coordination of the care site is in progress; WAIC: widely applicable information criterion.

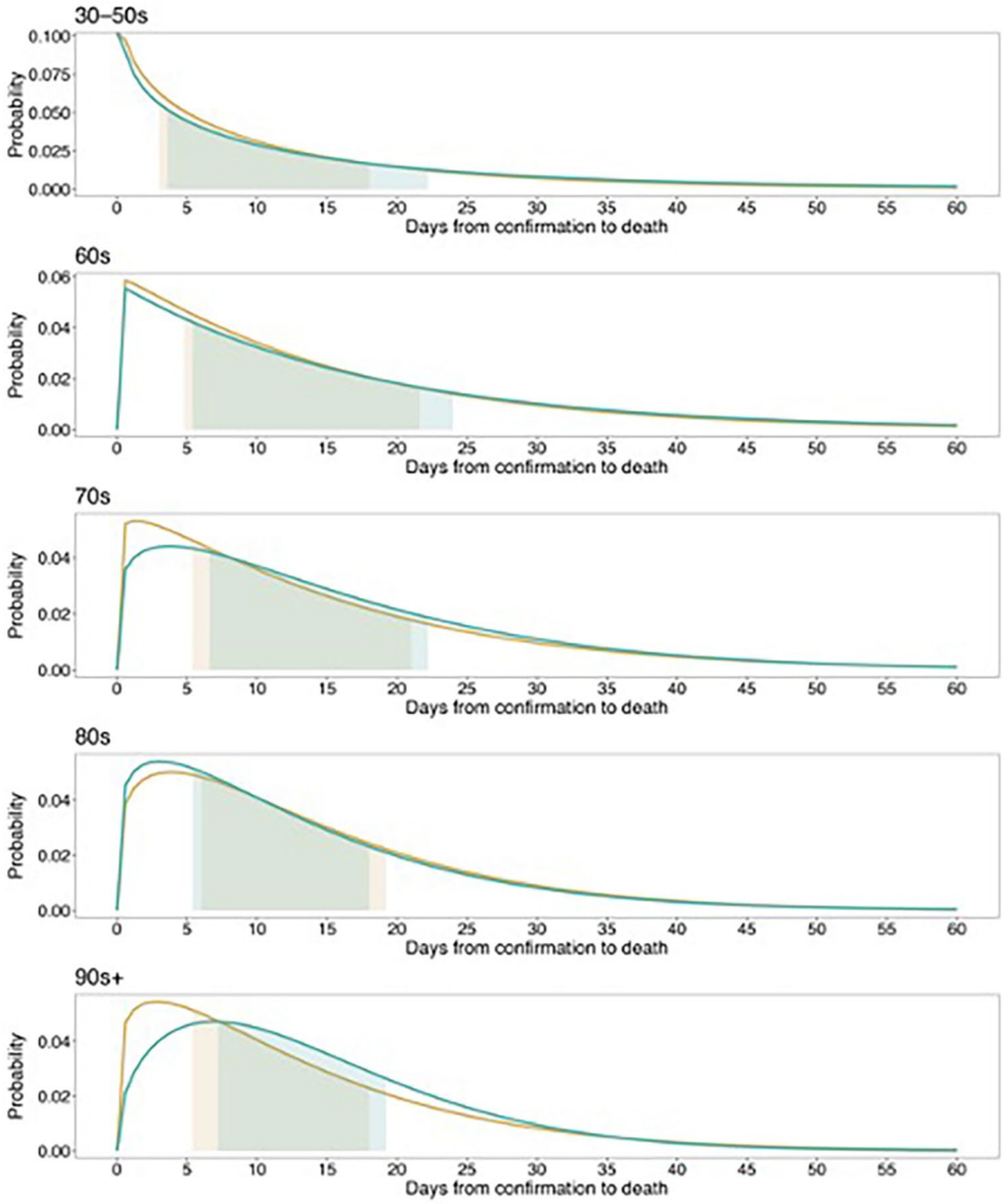

Appendix Figure 1. The distribution of days from confirmation to death fitted to a Weibull distribution according to age group. Each shade indicates the area between the first and third quartiles of the respective distribution.

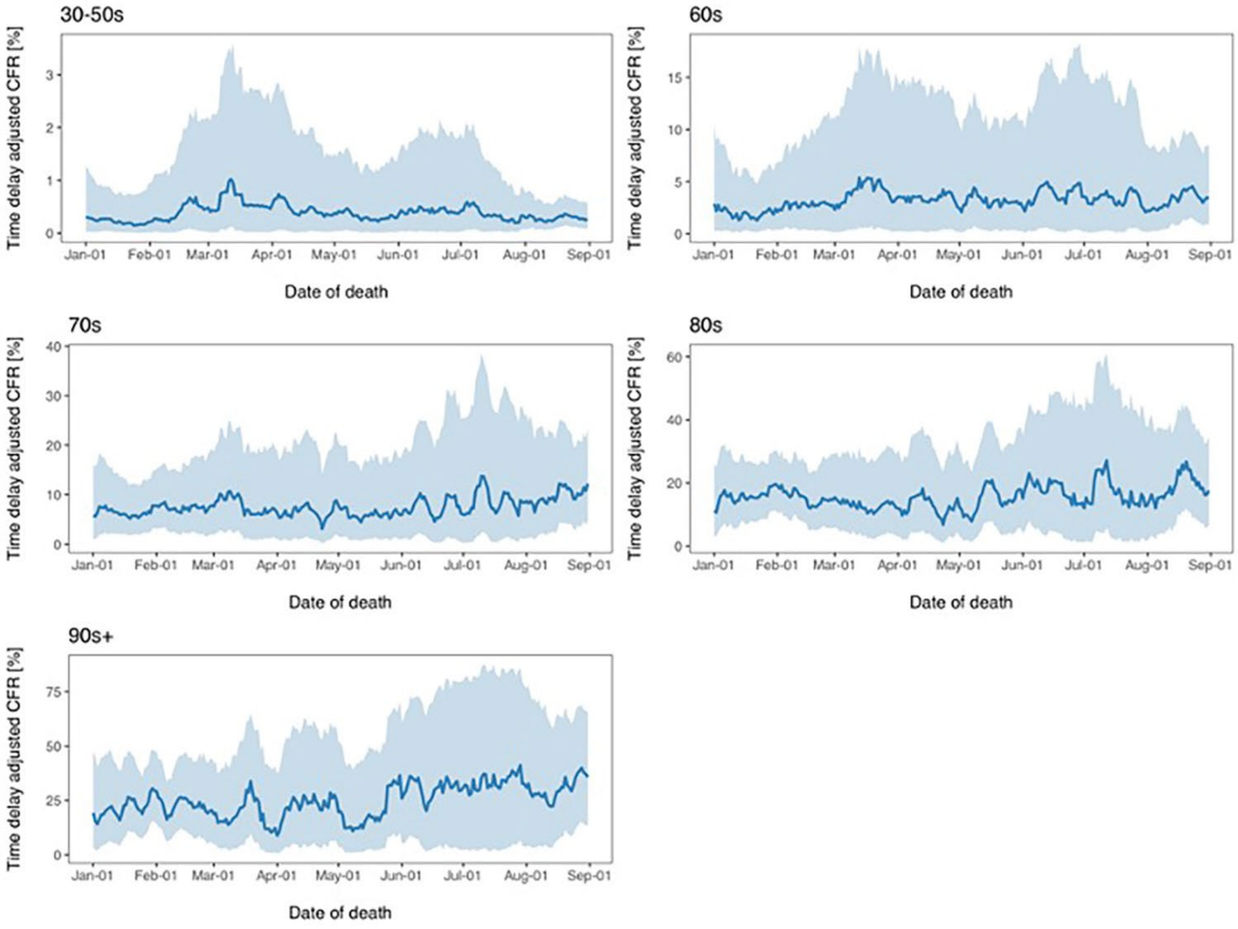

Appendix Figure 2. The estimated case fatality risk (CFR) among unvaccinated individuals according to age group, with shaded areas showing the 95% CrI.

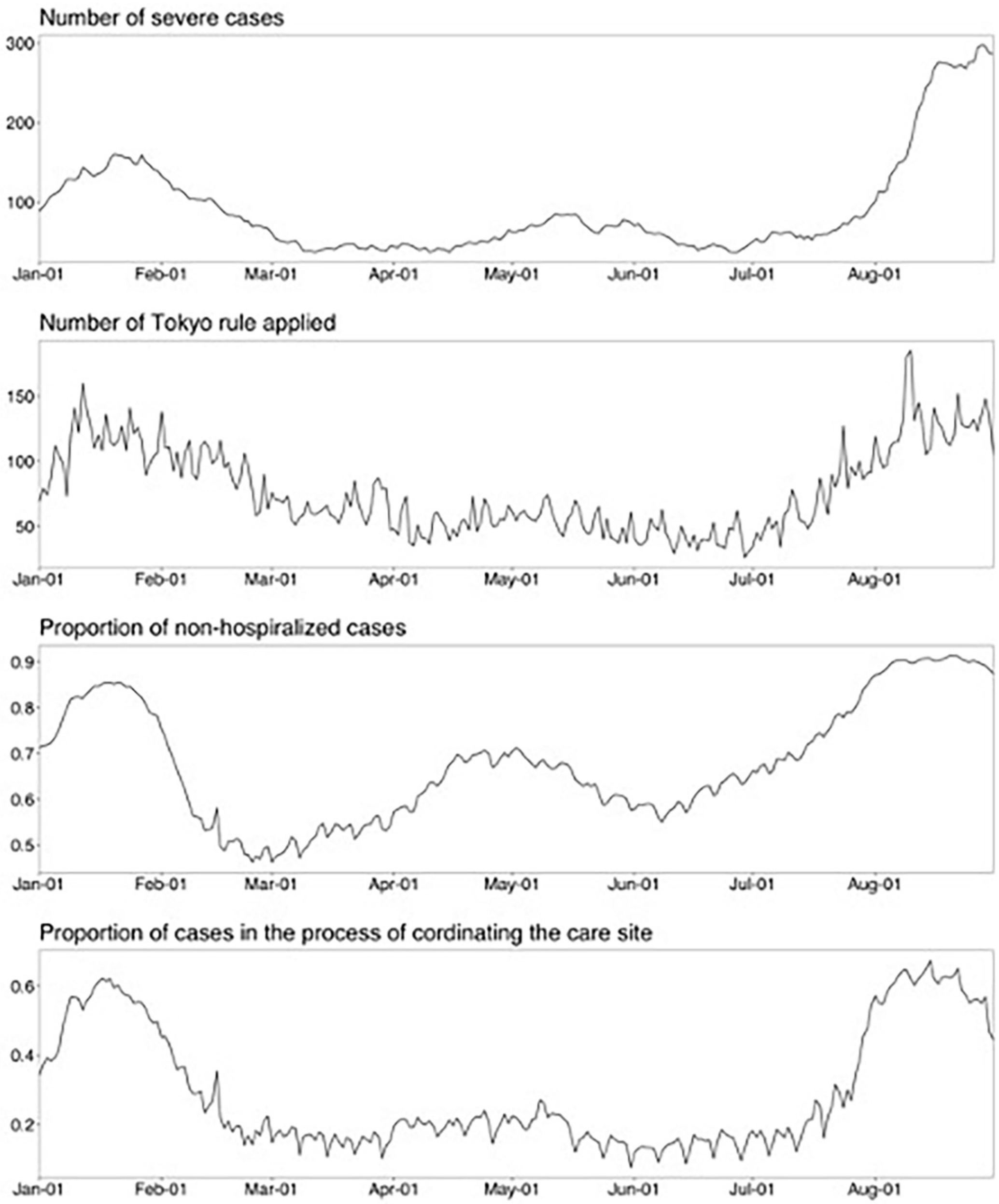

Appendix Figure 3. The trajectories of indicators for health care burden in Tokyo. Four indicators were used: 1) the number of severe COVID-19 cases, 2) the number of cases in which the Tokyo Rules applied, defined as cases in which the destination has not been determined within 20 minutes of the EMS request for or selection of five medical institutions to receive the patient, 3) the proportion of COVID-19 cases not hospitalized, and 4) the proportion of cases in which coordination of the care site was in progress.

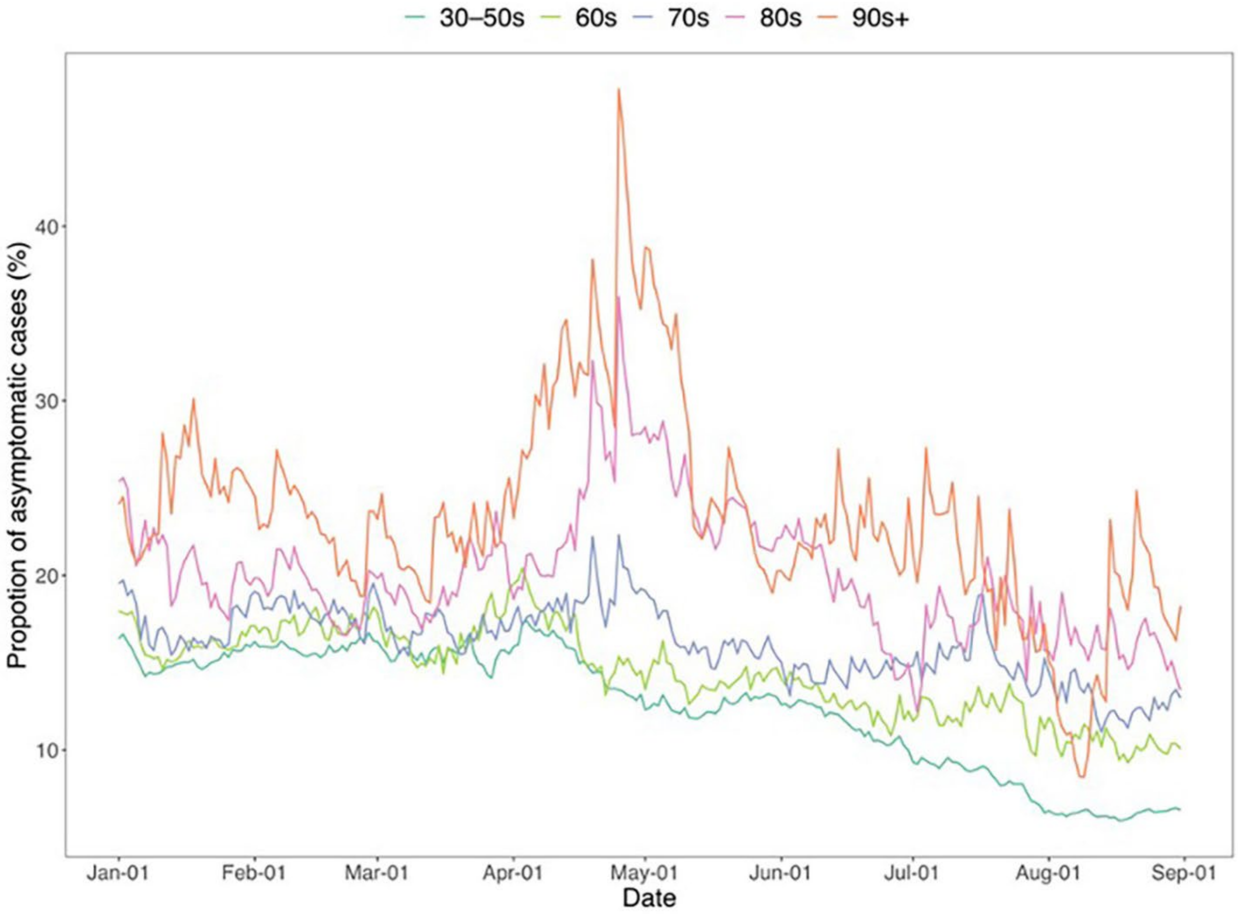

Appendix Figure 4. Proportion of asymptomatic cases among diagnosed individuals according to age group.

References

1. National Institute of Infectious Diseases. Estimation of the effectiveness of the new corona vaccine BNT162b2 (Pfizer/BioNTech) by applying a mathematical model to surveillance data (Report 1) [in Japanese][cited 2022 Jul 7]. https://www.niid.go.jp/niid/ja/2019-ncov/2484-idsc/10618-covid19-56.html

2. Watanabe S. Asymptotic equivalence of Bayes cross validation and widely applicable information criterion in singular learning theory. J Mach Learn Res. 2010;11:3571–94.
